# Association between TNF-Alpha (-857) Gene Polymorphism and Susceptibility to Tuberculosis 

**Published:** 2011-04-01

**Authors:** S Anoosheh, P Farnia, M Kargar

**Affiliations:** 1 Mycobacteriology Research Center, NRITLD, Shahid Beheshti University of Medical Sciences, Tehran, Iran; 2 Department of Microbiology, Islamic Azad University, Jahrom Branch, Jahrom, Iran

**Keywords:** Tuberculosis, TNF-α, Gene Polymorphism

## Abstract

**Background:**

TNF-α as a pro-inflammatory cytokine plays a key role in host defense against tuberculosis (TB). Presence of mutation in TNF-α gene can influence the effectiveness, performance and capability of immune responses against this infection. The Aim of this study was to investigate the frequency of TNF-α alleles and its relationship with susceptibility to TB and TNF-α gene variations.

**Methods:**

A case-control study was conducted and 103 healthy controls and 93 TB patients were enrolled. Genotype of TNF-238, TNF -244, TNF-308, TNF -857 and TNF-863 were distinguished using PCR-RFLP method.

**Results:**

TNF-857 and TNF-863 were in high frequency mutation regions in a population level, and a significant difference at TNF-857 was noticed between the two groups of case and control.

**Conclusion:**

Presence of mutation in TNF-857 region probably increases the host susceptibility to mycobacterial infection. Genotyping of these regions in combination with other factors can be used for screening of high risk persons. According to high distribution of mutations in TNF-857 and TNF-863 regions, further studies on association of these regions is suggested.

## Introduction

Tuberculosis (TB) is now among the most common infections which causes death in more than 2.000.000 patients annually.[1] Approximately one-third of world population is infected with Mycobacterium tuberculosis (M. tuberculosis), but the risk of developing disease ranges from 5-10%.[1][2] This suggest that besides the Mycobacteria itself, the host genetic factor may an important factor for differences in host susceptibility to TB. Recent studies have shown that there are individual differences in susceptibility to TB and everyone who is exposed to M. tuberculosis does not become infected. These differences might be due to host factors, especially host genes which will influence the susceptibility, severity and outcome of the disease.[2][3] Cell-mediated immunity (CMI) is considered as a defensive mechanism against intra-cellular pathogens similar to Leishmania and M. tuberculosis.[4] T-cells are the major effectors of CMI with the set of secreted cytokines that have a prominent role in the host defense against Mycobacterial infection.[4][5] Among these cytokines, tumor necrosis factor-α (TNF-α) has multiple roles in the defense and the pathological response to TB.[6] TNF-α has immunoregulatory properties and synergistic effects in addition to interferongamma (IFN-γ) in activation of macrophage and initiation of pro-inflammatory responses.[7][8] On the other hand, systemic spillover of TNF-α may account for unwanted inflammatory effects like fever and malaise. [8] TNF-α gene that encode the TNF-a cytokine is located within the major histocompatibility complex Class III (MHC class III) region at the short arm of chromosome 6 and its production is controlled both transcriptionally and post-transcriptionally.[9] Investigators have shown that mutation in this region would influence promoter activity or DNA and pre-mRNA conformation.[10] Among studied mutations, the single nucleotide polymorphisms (SNPs) are the most common genetic variation that occur at a frequency of approximately 1 in 1000 bp throughout the genome.[11] Polymorphisms in TNF-a gene have been associated in susceptibility to tuberculosis in different ethnic groups but the results have been inconclusive.[12][13][14][15][16][17][18][19][20][21][22] This work was conducted to study the -238, -244, 308, -857 and -863 TNF-α gene polymorphisms with respect to genotype and allele frequency distribution in patients with pulmonary tuberculosis among Iranian population to determine the association with tuberculosis.

## Materials and Methods

Ninety three pulmonary tuberculosis patients with positive sputum smears and cultures that referred to Masih Daneshvari Hospital, National Research Institute of Tuberculosis and Lung Disease, Tehran, Iran were selected for this study. Control group contained 103 healthy individuals who had no history of tuberculosis. The study was approved by Ethics Committee of National Research Institute of Tuberculosis and Lung Disease, Iran, and all subjects signed inform consents. Two milliliters of peripheral blood were collected in EDTA containing tube and stored at -4°C until DNA extraction.

Genomic DNA was extracted from peripheral blood leukocytes (PBLs), using the standard phenol– chloroform procedure with slight modifications.[23] The polymorphisms at TNF-238, -244, -308,-857 and -863 were determined. The primers and PCR conditions for each region were shown in Table 1 and Table 2. TNF-238 and -244 polymorphisms were determined by the polymerase chain reaction-restriction fragment length polymorphism (PCR-RFLP) method and the PCR product was digested directly with 10 U of Bgl II and Bsaj I restriction enzymes (Fermentas), respectively.[24] The TNF-308, -857 and -863 polymorphisms were also determined by PCR-RFLP method. We used Nco I as the restriction enzyme for TNF-308 and Tai I as the restriction enzyme for TNF-857 and TNF-863.[24][25] Digested PCR products were run on 8% polyacrylamide gel which was stained with silver-nitrate and digestion pattern was analyzed. The digest pattern of each polymorphism is illustrated in Table 3.

**Table 1:** PCR Conditions used for amplifying the TNF-α regions.

** TNF -238/-244 **		** TNF -308 **		** TNF -857 **		** TNF -863 **	
1 cycle	94° for 3 min	1 cycle	94° for 4 min	1 cycle	94° for 4 min	1 cycle	94° for 4 min
30 cycle	94° for 40 sec	30 cycle	94° for 60 sec	30 cycle	94° for 40 sec	30 cycle	94° for 45 sec
	53° for 40 sec		56° for 60 sec		56.5° for 40 sec		53° for 45 sec
	72° for 55 sec		72° for 60 sec		72° for 55 sec		72° for 60 sec
1 cycle	72° for 6 min	1 cycle	72° for 8 min	1 cycle	72° for 6 min	1 cycle	72° for 6 min

**Table 2:** The primers sequences used for amplifying the TNF-α regions.

** Regions **	** Primer sequences **
TNF -238/-244	5'- CCTCAAGGACTCAGCTTTCGT -3'
	5'- ACACTCCCCATCCTCCCACATC -3'
TNF -308	5'- AGGCAATAGGTTTTGAGGGCCAT -3'
	5'- TCCTCCCTGCTCCGATTCCG -3'
TNF -857	5'- GGCTCTGAGGAATGGGTTAC -3'
	5'- CCTCTACATGGCCCTGTCTAC -3'
TNF -863	5'- GGCTCTGAGGAATGGGTTAC -3'
	5'- CCTCTACATGGCCCTGTCTAC -3'

**Table 3:** Digest pattern of each SNP on polyacrylamide gel electrophoresis.

** SNP **		** PCR product Size **	** Enzyme **	** Digest pattern **
TNF-238	(G →A)	239 bp	Bgl II	A (216, 23 bp )
				G (239 bp )
TNF-244	(G →A)	239 bp	Bsaj I	G (169,78 bp )
				A (239 )
TNF-308	(G →A)	107 bp	Nco I	G (87, 20 bp )
				A (107 bp )
TNF-857	(C →T)	127 bp	Tai I	C (109, 18 bp )
				T (127 bp )
TNF-863	(C →A)	126 bp	Tai I	A (105, 21 bp )
				C (126 bp )

All statistical analyses were performed using SPSS software (version 16, Chicago, IL, USA). Chi-Square test was utilized to define any statistically significant differences among polymorphic allele or genotype between the two case and control groups. P values were calculated using Fisher Exact test (p<0.05 was considered statistically significant). The data were analyzed for their fitness to Hardy-Weinberg equilibrium.

## Results

The mean age was 50.04 years for patients and 38.75 years for controls, and the two groups were matched for gender. The mean age of cases was higher than controls but the difference was not significant. Genotype and allele frequencies in patients and healthy people were shown in Table 4.

**Table 4:** Genotype and allele frequencies in patients and healthy people

**SNP**		**Control**		**Patient**		***P*-value**
		**N**	**%**	**N**	**%**	
TNF-238	Genotype					NS^a^
	G/G	98	95.1	84	90.3	
	G/A	5	4.9	9	9.7	
	A/A	0	-	0	-	
	Allele frequency					NS
	G	201	97.6	177	95.2	
	A	5	2.4	9	4.8	
TNF-244	Genotype					NS
	G/G	103	100	93	100	
	G/A	0	-	0	-	
	A/A	0	-	0	-	
	Allele frequency					NS
	G	206	100	186	100	
	A	-	0	-	0	
TNF-308	Genotype					NS
	G/G	87	84.5	84	90.3	
	G/A	16	15.5	9	9.7	
	A/A	0	-	0	-	
	Allele frequency					NS
	G	190	92.2	177	95.2	
	A	16	7.8	9	4.8	
TNF-857	Genotype					0.001
	C/C	83	80.6	51	54.8	
	C/T	18	17.5	42	45.2	
	T/T	2	1.9	0	-	
	Allele frequency					0.002^b^
	C	184	89.3	144	77.4	
	T	22	10.7	42	22.6	
TNF-863	Genotype					NS
	C/C	74	71.8	69	74.2	
	C/A	29	28.2	21	22.6	
	A/A	0	-	3	3.2	
	Allele frequency					NS
	C	177	85.9	159	85.5	
	A	29	14.1	27	14.5	

^a^ NS: Nonsignificant

^b^ odds: 2.4, C.I 95% (1.3-4.4)

Frequency of alleles of TNF-238A, TNF-308A, TNF-857T, and TNF-863A in the control group were 2.4%, 7.8%, 10.7%, and 14.1% respectively, and 4.8%, 4.8%, 22.6%, and 14.5% for case group, respectively. No change was observed in the region of TNF-244. Comparison of genotypes between the control and case groups showed a significant difference at region of TNF-857 ; the frequency of C/T at region-857 in control and case groups was 17.5% and 45.2% respectively (p-value < 0.001, odds: 2.4; CI 95%: 1.3-2.4).

In regions of TNF-238, TNF-244 , TNF-308 and TNF-863, no significant relationship was observed between the two groups. The distribution of alleles of regions -238, -308, -857 and -863 in the control group showed a good fitness using the Hardy–Weinberg equilibrium.

## Discussion

Many single nucleotide polymorphisms are detected in the TNF-α gene region but most of the effective polymorphisms on TNF-α gene expression are those which are located in promoter region and has been altered levels of circulating TNF-α.[26][27] During recent years, the association between these polymorphisms and many illnesses including a wide range of infectious and autoimmune diseases as well as some prevalent cancers have been studied through which some relationships were defined concerning the susceptibility to diseases and the severity of them.[28][29][30] TNF-α is an important mediator in inflammatory responses and in mice deficient in TNF-α was shown to fail to form organized granuloma, which resulted in widespread dissemination of M. tuberculosis and rapid death of infected animals.[31] Previous studies on association between TNF-α gene polymorphisms and tuberculosis were limited and most of them focused only on the two regions -308 and -238 with actually different results.[3][5][12][13][14][15][16][17][18][19][20][21][22]

In this study, we found new association between TNF-857 and TB while the TNF-857 C/T genotype had significantly a higher frequency in TB patients. However, Delgado did not find any significant association between TB and TNF-857 in Cambodian population.[16] We also found no significant association between the TNF-238, TNF-308 polymorphisms with TB, which is consistent with some previous studies.[15][16][17][18][19] Selvaraj showed that none of the TNF-238 and TNF-308 polymorphisms were associated with pulmonary TB. Also, Ates did not notice any significant association between TNF-238, TNF-308 and TNF-376 polymorphisms and TB.[15][16][17][18][19] In this context, some researchers noted that the TNF-308 G/G significantly decreased among TB cases.[5][12][14] Bikmaeva reported that the frequency of allele TNF-308/A in tuberculosis patients was significantly higher than in controls.[5][12][14] Some studies considered the presence of TNF-238A as a risk factor for susceptibility to TB.[13][14] Correa reported that the TNF-238A allele was protective for autoimmunity but represented a susceptibility factor for TB.[13][14]

The associations between TNF-a production and TNF promoter polymorphisms are controversial. Wilson's study showed that TNF-308A is a much stronger transcriptional activator than TNF-308G in the human B cell line, and the TNF-308 polymorphisms have direct effects on TNF gene regulation.[32] However, Uglialoro's study showed that the TNF-308 polymorphisms did not affect the TNF gene expression in activated lymphocytes.[33] Furthermore, There were no significant association between TNF -863 polymorphisms and TB in this study, but this region showed high diversity of allele among studied population, which is similar to Delgado's results in combodia.[16] The polymorphisms at position TNF-244 could not be detected in the present study, which is consistent with Yen's study in Taiwan.[24]

These divergent findings may be due to ethnical differences in various populations that is why the studies that were performed in healthy controls showed allele diversities.[34][35][36][37][38] Generally, the single nucleotide polymorphisms in several candidate genes have been linked to relatively increased risk of TB.[28][29][30][39] Although, studies with higher number of cases might lead to more definite and reliable results regarding the relationship between TNF-α gene polymorphism and pulmonary tuberculosis. However, the importance of other genetical factors such as VDR (Vitamin D Receptor), and NRAMP1 (natural resistance-associated macrophage protein-1) should not be ignored.

We can conclude that TNF-857C/T genotype may be considered as a genetic marker that influences the susceptibility to tuberculosis in studied Iranian populations. Therefore, more comprehensive studies on -857 and -863 allele diversity of TNF gene is necessary.
